# Ultrasonographic evaluation of adrenal glands in clinically healthy Korean raccoon dogs (*Nyctereutes procyonoides koreensis*)

**DOI:** 10.3389/fvets.2025.1692326

**Published:** 2025-12-12

**Authors:** Jinkyung Park, Myeongsu Kim, Jae-Ik Han, Kichang Lee, Hakyoung Yoon

**Affiliations:** 1 Department of Veterinary Medical Imaging, College of Veterinary Medicine, Jeonbuk National University, Iksan, Republic of Korea; 2 Laboratory of Exotic and Wild Animal Medicine, College of Veterinary Medicine, Jeonbuk National University, Iksan, Republic of Korea

**Keywords:** corticomedullary distinction, adrenal ultrasonography, reference values, diagnostic imaging, wild canid, wildlife medicine

## Abstract

**Introduction:**

Ultrasonographic evaluation of the adrenal glands is a valuable tool for detecting structural abnormalities during clinical assessment. However, no species-specific sonographic reference values have been established for Korean raccoon dogs (*Nyctereutes procyonoides koreensis*), limiting the application of this modality in clinical and wildlife settings.

**Methods:**

Sagittal ultrasonographic images from 38 clinically healthy Korean raccoon dogs were retrospectively analyzed. Adrenal length and cranial and caudal pole heights were measured bilaterally, with each parameter obtained three times per gland and the median value used for analysis. Gland morphology and corticomedullary distinction were visually assessed, and statistical tests were performed to evaluate differences by sex, body weight, and laterality.

**Results:**

All adrenal glands were clearly visualized without sedation and consistently exhibited a peanut-shaped morphology. Mean adrenal lengths were 14.12 ± 2.25 mm on the left and 14.33 ± 1.97 mm on the right. Cranial and caudal pole heights were 3.10 ± 0.44 and 3.26 ± 0.33 mm, respectively, on the left, and 3.19 ± 0.61 and 3.50 ± 0.52 mm, respectively, on the right. Within each gland, the caudal pole was significantly greater in height than the cranial pole. In addition, the right caudal pole height was significantly greater than the left (*p* = 0.002). No significant associations with sex or body weight were found. Corticomedullary boundaries were visible in 94.7% of left and 76.3% of right adrenal glands.

**Conclusion:**

This study provides species-specific ultrasonographic reference values for Korean raccoon dogs, supporting improved interpretation in clinical and conservation medicine.

## 1 Introduction

The Korean raccoon dog (*Nyctereutes procyonoides koreensis*) is a wild canid species endemic to the Korean Peninsula. The recent steady population increase, due to changes in habitat and a reduction in natural predators (1), has led to a rise in clinical admissions and greater interest in wildlife medicine and diagnostic imaging. As a result, several imaging-based studies, involving radiographic, echocardiographic, and abdominal ultrasonographic evaluations (2–4), have been conducted in this species. However, no study has yet established sonographic reference values for the adrenal glands.

Non-invasive abdominal ultrasonography is a widely used screening tool in rescued wildlife patients. Nonetheless, the lack of species-specific reference values for internal organs critically limits the accurate interpretation of the acquired images. Adrenal glands are presumed to be situated ventromedially to the cranial pole of the kidneys in raccoon dogs, as in domestic dogs (5–8). In canine studies, consistent ultrasonographic visualization of the adrenal glands is technically difficult due to their small size, deep anatomical position, and variable morphology. These limitations, combined with interobserver variation, reduce the reliability of adrenal structural assessment in clinical settings (9–11).

Previous studies in dogs have shown that adrenal morphology varies by laterality, with the left gland typically peanut-shaped and the right gland exhibiting a more variable configuration (12, 13). Adrenal size is also variable, and has been reported to correlate with factors such as body weight, age, and breed, leading to the development of side-specific and weight-based reference intervals (12, 14–16). Ultrasonographic studies conducted in cats have revealed species-specific characteristics of the adrenal glands, which are generally smaller than those found in dogs, ovoid in shape, and uniformly hypoechoic with poor corticomedullary distinction (6, 8). The interspecies differences observed between dogs and cats highlight the importance of establishing independent sonographic reference values for wildlife species, such as the Korean raccoon dog (6, 8, 17). Given the known physiological and genetic differences between Korean raccoon dogs and domestic dogs, reference values derived for dogs or cats may have limited relevance (1, 18).

Therefore, this study aimed to establish baseline ultrasonographic measurements of adrenal length and cranial and caudal pole heights, and to determine the morphological features, corticomedullary distinction, and laterality of adrenal glands in clinically healthy Korean raccoon dogs. The findings are expected to provide foundational data to support more accurate clinical assessments and contribute to the establishment of species-specific diagnostic criteria in wildlife imaging.

## 2 Materials and methods

### 2.1 Ethical approval

This study was approved by the Institutional Animal Care and Use Committee of Jeonbuk National University (Approval No. NON2025-003). All procedures involving animals were conducted in accordance with institutional guidelines and applicable local regulations.

### 2.2 Animals and study design

This retrospective study included data obtained from Korean raccoon dogs (*Nyctereutes procyonoides koreensis*) rescued and admitted to the Wildlife Medical Center at Jeonbuk National University between November 2021 and April 2025. Upon admission, each animal underwent a physical examination, complete blood count, serum biochemistry, infectious disease screening using the SNAP 4Dx® Test (IDEXX Laboratories, Saint Helens, UK), canine distemper virus and canine parvovirus antigen testing, and abdominal ultrasonography.

Animals were included if they exhibited no clinical signs of systemic illness, had no history of corticosteroid administration, and showed no significant abnormalities on diagnostic testing. Mild and transient elevations in alanine aminotransferase or alkaline phosphatase were considered acceptable when unaccompanied by additional clinical, hematologic, or ultrasonographic abnormalities, as such fluctuations are commonly observed in recently rescued wildlife and are widely regarded as physiologic stress responses rather than indicators of hepatobiliary disease.

Juvenile animals were excluded as the adrenal glands of growing individuals undergo anatomical and endocrine maturation that may affect size and ultrasonographic appearance. Because chronological age is rarely available for rescued Korean raccoon dogs, juvenile status was assessed using physical maturity indicators routinely applied in wildlife rescue centers, including body weight, dentition stage, and skeletal maturity on physical examination or radiographs.

Additional exclusion criteria included clinical signs suggestive of systemic illness (e.g., dehydration, anemia, fever, diarrhea, or marked lethargy), hematologic or infectious abnormalities, and ultrasonographic abnormalities, including irregular contour or discrete hyperechoic nodules. These criteria were applied to ensure that the resulting measurements reflected baseline adrenal morphology rather than alterations associated with illness or stress-related endocrine activation.

Of the 46 animals initially evaluated, 4 were excluded due to age and 4 due to systemic or adrenal abnormalities. Finally, 38 animals (22 males and 16 females; mean body weight: 3.88 ± 0.64 kg) were included in the subsequent analyses.

### 2.3 Ultrasonographic evaluation

Ultrasonographic examinations were performed using two ultrasound systems (Aplio 300 and Aplio i800; Canon Medical Systems, Ōtawara, Japan), each equipped with a 12 MHz linear transducer. All scans were conducted by a senior veterinary radiologist with more than 10 years of clinical experience using a standardized scanning protocol (9, 10). Adrenal measurements were performed after image acquisition by a radiology resident with two years of training, using electronic calipers. To minimize measurement errors, each parameter was measured three times per adrenal gland; the median of these three measurements was used for statistical analysis (10). Animals were manually restrained in dorsal recumbency without sedation and examined during spontaneous respiration (6, 8). Both adrenal glands were evaluated in the sagittal plane.

**Figure 1:**
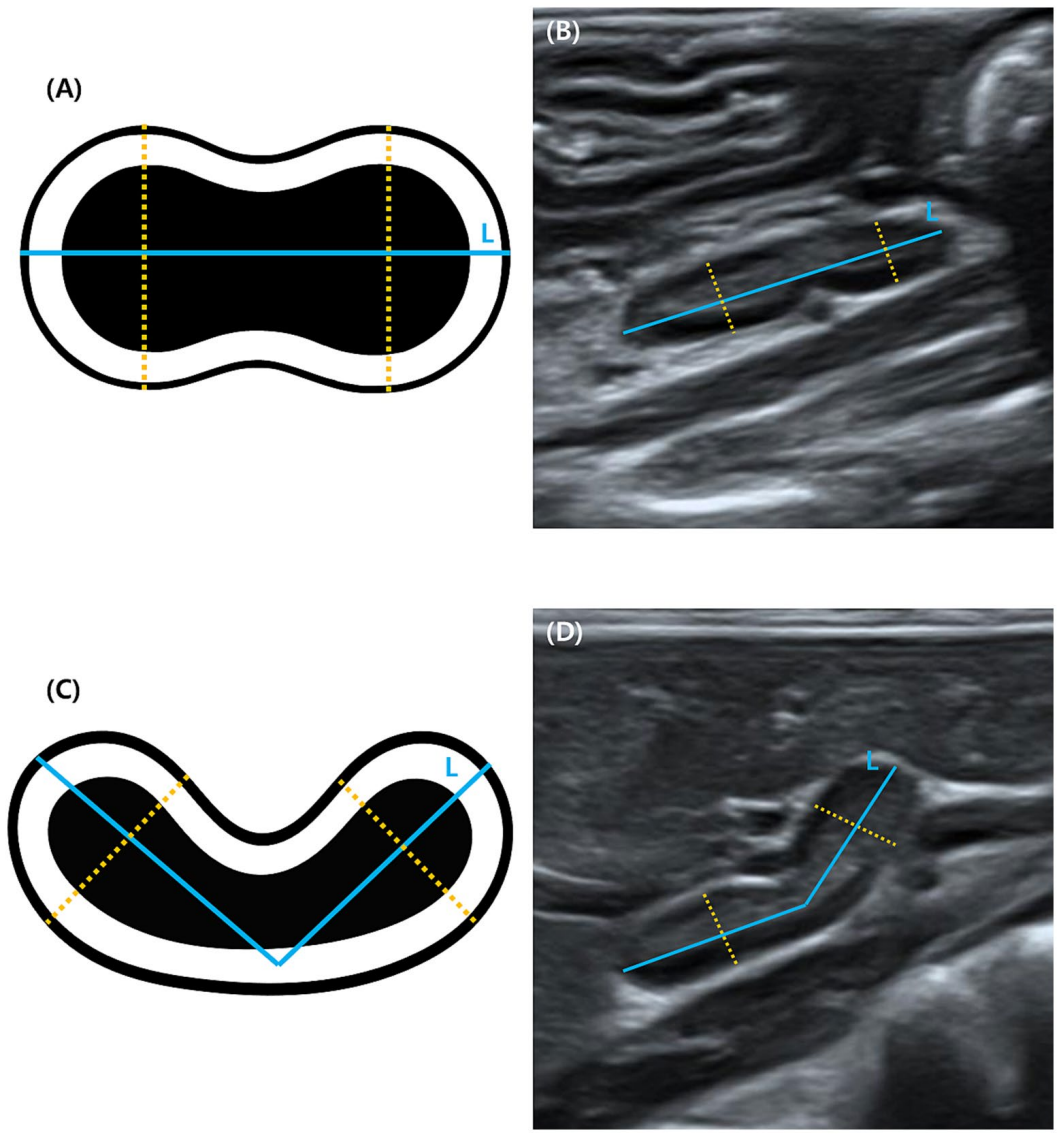
Schematic diagrams and ultrasonographic images of adrenal glands in the sagittal plane. **(A)** In straight glands, adrenal length (L) was measured from outer edge to outer edge along the craniocaudal axis. Cranial pole (CP) and caudal pole (CaP) heights were measured from leading edge to trailing edge, perpendicular to the long axis. **(B)** Longitudinal ultrasonographic image of a straight adrenal gland in a clinically healthy Korean raccoon dog. **(C)** In curved glands, adrenal length (L) was measured by tracing the outer margin from the cranial to the caudal pole. Cranial pole (CP) and caudal pole (CaP) heights were measured as the maximum dorsoventral distances, each taken perpendicular to the local curvature at the respective pole. **(D)** Longitudinal ultrasonographic image of a curved adrenal gland in a clinically healthy Korean raccoon dog. Measurement lines are shown as a curved solid line (L) and dotted lines (CP, CaP).

Three morphometric parameters were measured (12): [1] total adrenal length, defined as the maximum craniocaudal distance between the cranial and caudal poles, measured from outer edge to outer edge along the longitudinal axis; [2] cranial pole height; and [3] caudal pole height. The latter two parameters were determined by placing the caliper perpendicular to the long axis of the gland to measure the maximum dorsoventral thickness at each pole from leading edge to trailing edge. A schematic diagram and representative ultrasonographic image of a straight adrenal gland are presented in Figures 1A,B, respectively. In curved adrenal glands, the length was measured by tracing the outer contour from the cranial to the caudal pole (11). Cranial and caudal pole heights were measured as the maximum dorsoventral distances, each taken perpendicular to the local curvature at the respective pole. These measurements account for the natural shape of the gland, rather than imposing a straight axis. This method is illustrated in Figures 1C,D.

In addition to morphometric assessment, two qualitative parameters were evaluated: corticomedullary distinction and glandular morphology. Corticomedullary distinction was graded visually as either distinct or indistinct based on the visibility of a hyperechoic medulla surrounded by a relatively hypoechoic cortex (6, 8, 12). Glandular morphology was classified into three types based on the adrenal contour in sagittal view: peanut-shaped, in which both poles were wider than the middle portion; oval-shaped, characterized by a smooth and rounded outline without polar tapering; and V-shaped, characterized by a sharp indentation or kink at the cranial pole (12, 14, 18). In the absence of a standardized classification even in dogs, we adapted previously reported morphological types (12, 18) and evaluated gland shape by visual inspection to ensure consistency.

### 2.4 Statistical analysis

Statistical analyses were conducted using SPSS software (version 20.0; IBM Corp., Armonk, NY, USA). The normality of continuous variables was assessed using the Shapiro–Wilk test. All data followed a normal distribution and are therefore presented as mean ± SD, with 95% confidence intervals (CIs).

Paired t-tests were used to compare left and right adrenal gland measurements, and independent t-tests were used to evaluate sex differences; equality of variances was verified with Levene’s test. Pearson correlation analysis was performed to assess the relationships between adrenal dimensions and body weight.

Statistical significance was set at *p* < 0.05. No statistical test was conducted for qualitative parameters such as corticomedullary distinction due to the categorical nature of the data and the limited number of cases with indistinct boundaries.

## 3 Results

### 3.1 Adrenal gland visualization, measurement, and shape classification

All adrenal glands in the 38 raccoon dogs (*n* = 76) were successfully visualized in the sagittal plane. Each gland showed clear delineation of the cranial and caudal poles, particularly on the left side. Visualization of the right adrenal gland was occasionally limited by interference from intestinal gas or adjacent anatomical structures. No sex-based differences in adrenal visibility were observed. Adrenal measurements are presented in Table 1.

**Table 1:** Adrenal gland measurements in clinically healthy Korean raccoon dogs (*n* = 38).

Parameter	Left adrenal gland	Right adrenal gland
Adrenal length (mm)	14.12 ± 2.25 (13.38–14.86)	14.33 ± 1.97 (13.68–14.97)
Cranial pole height (mm)	3.10 ± 0.44 (2.95–3.24)	3.19 ± 0.61 (2.99–3.39)
Caudal pole height (mm)	3.26 ± 0.33 (3.15–3.37)	3.50 ± 0.52 (3.33–3.67)

Values are presented as mean ± SD with 95% confidence intervals.

All visualized adrenal glands exhibited a uniform peanut-shaped morphology on sagittal ultrasonographic evaluation (Figures 1B,D), characterized by narrowing at the midbody and symmetrical thickening at both poles. No oval- or V-shaped glands were observed.

### 3.2 Corticomedullary differentiation

**Figure 2:**
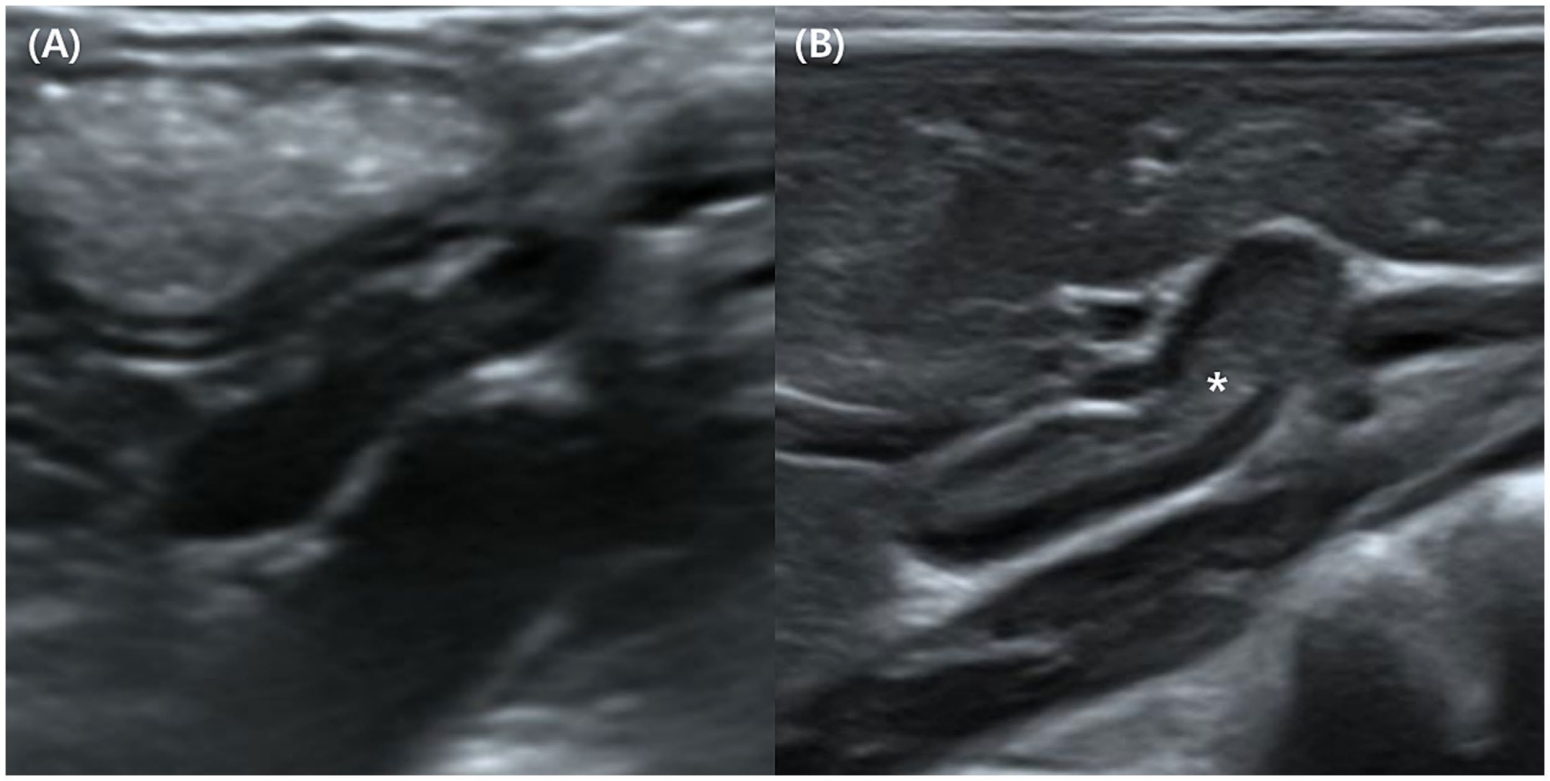
Longitudinal ultrasonographic images of adrenal glands in Korean raccoon dogs. **(A)** An adrenal gland exhibiting an indistinct corticomedullary boundary with homogeneous echogenicity. **(B)** An adrenal gland exhibiting a distinct corticomedullary boundary with a hypoechoic cortex and hyperechoic (or isoechoic) medulla marked by an asterisk (*).

The corticomedullary boundary was clearly visualized in 36 of 38 left adrenal glands (94.7%) and 29 of 38 right adrenal glands (76.3%). Indistinct or poorly defined boundaries were therefore more frequently observed in the right (9/38, 23.7%) than in left (2/38, 5.3%) adrenal glands (Figure 2). However, no statistical analysis was performed due to the qualitative nature of this assessment.

### 3.3 Sex-based differences in adrenal measurements

As shown in Table 2, no significant differences were observed between males (*n* = 22) and females (*n* = 16) for any of the measured parameters (all *p* > 0.05). The mean left adrenal length was 13.80 ± 1.86 mm in males and 14.56 ± 2.71 mm in females (*p* = 0.34). The left cranial pole height was 3.15 ± 0.48 mm in males and 3.02 ± 0.37 mm in females (*p* = 0.37), while the left caudal pole height measured 3.28 ± 0.29 mm in males and 3.24 ± 0.38 mm in females (*p* = 0.70). The mean right adrenal length was 14.27 ± 1.99 mm in males and 14.41 ± 2.01 mm in females (*p* = 0.84). Similarly, right cranial (males: 3.15 ± 0.63 mm, females: 3.24 ± 0.60 mm; *p* = 0.66) and caudal (males: 3.52 ± 0.58 mm, females: 3.46 ± 0.44 mm; *p* = 0.72) pole heights did not differ significantly between sexes. These results indicate that adrenal measurements can be applied irrespective of sex in clinically healthy Korean raccoon dogs.

**Table 2:** Adrenal gland measurements according to sex.

Parameter	Males (*n* = 22)	Females (*n* = 16)	*p* value
Left adrenal length (mm)	13.80 ± 1.86 (12.97–14.62)	14.56 ± 2.71 (13.12–16.01)	0.34
Left cranial pole height (mm)	3.15 ± 0.48 (2.94–3.36)	3.02 ± 0.37 (2.83–3.22)	0.37
Left caudal pole height (mm)	3.28 ± 0.29 (3.15–3.41)	3.24 ± 0.38 (3.03–3.44)	0.70
Right adrenal length (mm)	14.27 ± 1.99 (13.39–15.15)	14.41 ± 2.01 (13.34–15.48)	0.84
Right cranial pole height (mm)	3.15 ± 0.63 (2.88–3.43)	3.24 ± 0.60 (2.93–3.56)	0.66
Right caudal pole height (mm)	3.52 ± 0.58 (3.26–3.78)	3.46 ± 0.44 (3.23–3.70)	0.72

Values are presented as mean ± SD with 95% confidence intervals. Independent *t*-tests were used to compare the groups.

### 3.4 Correlation between adrenal measurements and body weight

No significant correlations were found between body weight and either the left (*r* = 0.08, *p* = 0.64) or right (*r* = 0.10, *p* = 0.56) adrenal gland length. Similarly, no significant correlations were observed between body weight and left (cranial: *r* = −0.06, *p* = 0.74; caudal: *r* = −0.15, *p* = 0.36) or right (cranial: *r* = −0.28, *p* = 0.09; caudal: *r* = −0.16, *p* = 0.33) pole height. These findings indicate that adrenal size is independent of body weight in clinically healthy Korean raccoon dogs.

### 3.5 Comparison of left and right adrenal glands

**Figure 3:**
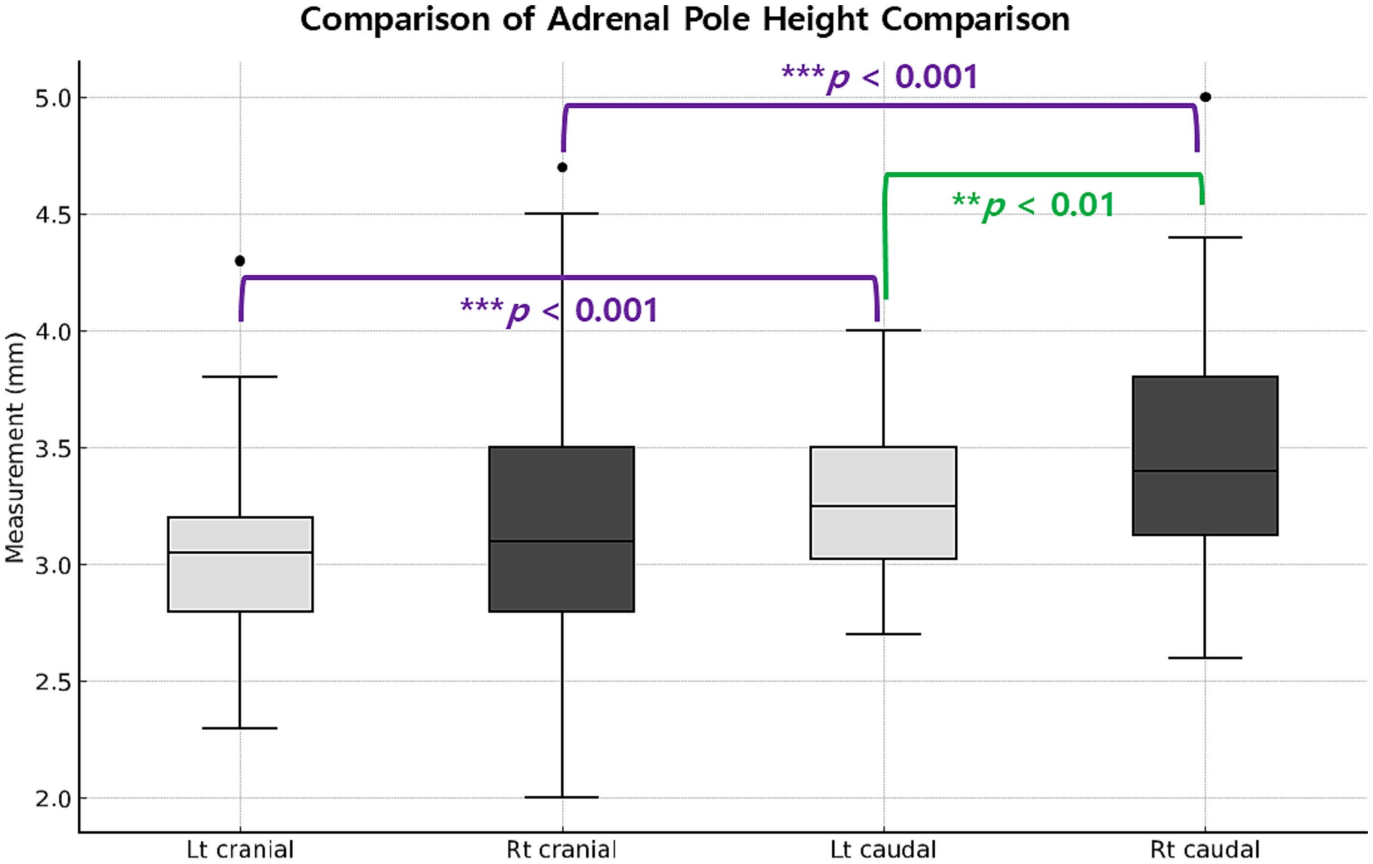
Comparison of cranial and caudal pole heights in the left and right adrenal glands. Boxes represent the interquartile range (Q1–Q3), with horizontal lines indicating the medians. Whiskers extend to 1.5 × interquartile range; individual outliers are shown as dots. Within each gland, the caudal pole was significantly greater than the cranial pole (***p < 0.001). Between glands, the right caudal pole was significantly greater than the left (*p* = 0.002), whereas cranial pole height did not differ significantly between sides.

The mean lengths of the left and right adrenal glands were 14.12 ± 2.25 and 14.33 ± 1.97 mm, respectively, with no significant difference between the two (*p* = 0.65). Similarly, no significant difference was detected in the cranial pole height between the left (3.10 ± 0.44 mm) and right (3.19 ± 0.61 mm) adrenal glands (*p* = 0.43). Within each gland, the caudal pole height was significantly greater than the cranial pole height (left: *p* < 0.001; right: *p* < 0.001). Moreover, the caudal pole height was significantly greater in the right adrenal gland (3.50 ± 0.52 mm) than in the left adrenal gland (3.26 ± 0.33 mm) (*p* = 0.002; Figure 3). Thus, the adrenal glands were largely symmetrical in length and cranial pole height, but showed consistent intra-gland asymmetry (caudal > cranial) and an inter-gland asymmetry with a greater caudal pole on the right side.

## 4 Discussion

Adrenal ultrasonography in Korean raccoon dogs resulted in consistent and reproducible imaging outcomes under standardized conditions, supporting the establishment of species-specific reference values. All adrenal glands were clearly visualized in the sagittal plane without sedation, enabling the accurate measurement of adrenal length and pole heights. This high visualization rate contrasts with earlier studies conducted in dogs, which noted frequent difficulties in detecting normal adrenal glands (19), and aligns with studies reporting reliable identification of adrenal glands under optimized conditions (20).

Morphologically, all adrenal glands imaged in the present study exhibited a peanut-shaped configuration, with no observed variants. In contrast, various morphologies, including fusiform, comma-shaped, and bean-shaped, have been reported as normal variants in domestic dogs (12–14, 18). The absence of such variability in Korean raccoon dogs suggests a high degree of species-specific morphological consistency, and may serve as a reference for detecting pathological changes. Interestingly, the overall sonographic appearance of adrenal glands in Korean raccoon dogs (small, peanut shaped, and sometimes with indistinct corticomedullary boundaries, particularly on the right) resembles that of domestic cats, whose glands are uniformly hypoechoic with limited internal architectural differentiation (6–8). This interspecies similarity, and the interspecies differences between Korean raccoon dogs and domestic dogs (12, 14), further supports the need for independent reference values for Korean raccoon dogs, to avoid a reliance on canine indices.

The corticomedullary boundary was clearly identified in most adrenal glands, but less frequently on the right side. This reduced visibility may be attributable to anatomical position, scanning depth, thoracic conformation, or interference from adjacent abdominal organs (10, 18, 21, 22). Previous canine studies have shown that imaging angle and probe orientation influence detection (9, 12, 14). As in dogs, such technical and anatomical factors should be considered before interpreting the absence of clear corticomedullary definition as pathological (13, 19).

No statistically significant differences were found in any of the adrenal gland parameters between males and females, and no significant correlations were identified between adrenal measurements and body weight. These findings contrast with previous canine studies in which adrenal size was shown to increase with body weight (12, 15, 16), and indicate that a single set of reference values may be applicable to adult Korean raccoon dogs, irrespective of sex and weight. The mean adrenal length in this study was 14.23 ± 2.11 mm, comparable to the left adrenal glands of small-breed dogs (< 5 kg; 13.8 mm; range: 12.4–15.9 mm) but greater than the right adrenal glands of small-breed dogs (11.8 mm; range: 10.1–12.9 mm) (14, 21), suggesting species-specific laterality. However, adrenal length is susceptible to measurement variability and positional differences. In contrast, left and right adrenal gland caudal pole heights were 3.26 ± 0.33 mm and 3.50 ± 0.52 mm, respectively, in the present study, aligning closely with previously reported caudal pole height in small-breed dogs (3.7 ± 0.6 mm; range: 2.4–5.4 mm) (12), regardless of body weight. This stability across species and size categories suggests that caudal pole height may serve as a more reliable morphometric parameter than adrenal length for clinical use.

In the present study, both adrenal glands showed consistent intra-gland asymmetry, with the caudal pole height being significantly greater than the cranial pole height on both sides. In addition, an inter-gland asymmetry was observed, as the right caudal pole height was significantly greater than the left. In contrast, adrenal length and cranial pole height did not differ between sides. Similar mild laterality has also been described in dogs, where such variations are regarded as normal anatomical features rather than pathological changes (12, 14, 18). Importantly, the present study provides quantitative data on cranial pole height, which has been rarely reported in canine studies, thereby offering a more comprehensive morphometric assessment of adrenal gland size and shape in Korean raccoon dogs. Despite standardized imaging protocols with triplicate measurements by a single experienced examiner, cranial pole identification remained more challenging on the right side, consistent with previous canine studies that attributed right-sided difficulty to anatomical and positional factors (9, 15). The observed predominance of the caudal pole, particularly on the right side, may reflect species-specific laterality in Korean raccoon dogs. Such asymmetry should be considered when establishing reference values to avoid misinterpretation, especially in distinguishing normal variation from true pathological enlargement.

In the present study, individual morphometric parameters in raccoon dogs showed both similarities and differences compared to reported values in dogs and cats (6–8). Adrenal length and cranial pole height in Korean raccoon dogs were broadly comparable to small-breed canines (8), whereas interspecies variations were observed in terms of caudal pole height and corticomedullary distinction (6, 14). These interspecies differences indicate that no single parameter can fully characterize adrenal morphology in Korean raccoon dogs. In dogs, adrenal length alone may be insufficient to detect subtle or early enlargement of the adrenal gland; pole height and corticomedullary distinction offer additional diagnostic sensitivity (12, 16). Incorporating multiple morphometric variables can also improve diagnostic accuracy and reduce interobserver variability (6, 9, 10, 12). Therefore, a combined assessment of adrenal length, cranial and caudal pole heights, and corticomedullary distinction is recommended to account for anatomical variation, minimize handling, and avoid sedation; these factors are critical for both clinical evaluation and wildlife conservation.

This study has several limitations. First, the relatively small sample size may not fully represent the anatomical variability of the broader Korean raccoon dog population. Future larger-scale studies are warranted to establish more robust reference ranges.

Second, histopathological confirmation and endocrine function testing were not performed, limiting the correlation between sonographic corticomedullary distinction and true structural or functional integrity. Although corticomedullary visualization was frequently achieved in this study, previous canine research (23, 24) has linked loss of distinction to histologically confirmed adrenocortical atrophy or neoplasia, as well as reduced gland thickness following adrenocorticotropic hormone stimulation in hypoadrenocorticism (23). Future studies incorporating hormonal assessment and histological confirmation are needed to clarify the diagnostic significance of these ultrasonographic findings, particularly in light of potential species-specific anatomical differences not fully addressed by canine-derived references.

Third, adrenal gland volume was not assessed. While linear measurements are practical in ultrasonography, they are subject to operator-dependent variability (9, 10) and may not fully reflect true gland size (11). Computed tomography-based volumetric analysis has been shown to correlate strongly with body weight in dogs, particularly when using automated segmentation models (26). In future research, volumetric approaches should be considered to provide a more comprehensive and operator-independent evaluation of adrenal morphology.

Despite these limitations, the present results provide foundational data for establishing species-specific ultrasonographic criteria in Korean raccoon dogs, with potential applications in both clinical diagnosis and wildlife health assessment, where objective and reproducible evaluations are critical.

## 5 Conclusion

This study establishes the first ultrasonographic reference values for adrenal gland measurements and morphology in clinically healthy Korean raccoon dogs. Adrenal glands consistently exhibited a peanut-shaped contour and were readily visualized in the sagittal plane without sedation. The absence of significant variation according to sex or body weight supports the applicability of a single set of reference ranges for this species. These baseline data are expected to enhance diagnostic precision in veterinary imaging and provide a framework for species-specific assessment in wildlife medicine.

## Data Availability

The raw data supporting the conclusions of this article will be made available by the authors, without undue reservation.
